# Effects of N-Acetyl-L-Cysteine on Serum Indices and Hypothalamic AMPK-Related Gene Expression Under Chronic Heat Stress

**DOI:** 10.3389/fvets.2022.936250

**Published:** 2022-06-15

**Authors:** Zhengqing Li, Yulan Zhao, Yu Zhuang, Zheng Xu, Cong Wu, Ping Liu, Guoliang Hu, Guyue Li, Wei Chen, Xiaona Gao, Xiaoquan Guo

**Affiliations:** ^1^Jiangxi Provincial Key Laboratory for Animal Health, Institute of Animal Population Health, College of Animal Science and Technology, Jiangxi Agricultural University, Nanchang, China; ^2^Department of Mathematics and Statistics, Wright State University, Dayton, OH, United States

**Keywords:** hypothalamus, heat stress, AMPK pathway, appetite, N-acetyl-l-cysteine

## Abstract

This study aims to investigate the effect of heat stress on the physiological metabolism of young laying hens and whether N-acetyl-l-cysteine (NAC) can effectively alleviate heat stress. 120 Hy-Line Brown laying hens aged 12 weeks were randomly divided into four groups: the control group (fed on basal diet under thermal neutral condition), HS group (fed on basal diet under heat stress condition), CN group (fed on the basic meal supplemented with 1,000 mg NAC per kg under thermal neutral condition), and HS+N group (fed on the basic meal was supplemented with 1000 mg NAC per kg under heat stress condition). The HS and HS+N groups were exposed to 36 ± 1°C for 10 h/day. The effects of NAC on the changes of serum concentrations of T3, T4, and CORT and hypothalamic gene and protein expressions induced by heat stress were measured. Results showed that heat stress upregulated the contents of T3, T4, and CORT, while NAC reduced the contents of T3, T4, and CORT. In addition, NAC downregulated AgRP expression, while upregulated the expression of POMC. Moreover, the expressions of AMPKα1, LKB1, and CPT1 were inhibited by NAC, while the expressions of AKT1, ACC, GPAT, and PPARα were increased after NAC treatment, and HMGR did not change significantly. Western blot and comprehensive immunofluorescence section of AMPK in the hypothalamus showed that NAC attenuated the activity of AMPK. In conclusion, NAC can enhance the resistance of laying hens to heat stress by alleviating the metabolic disorders of serum T3, T4, and CORT induced by heat stress, inhibiting the activation of the AMPK pathway and regulating the expression of appetite-related genes in the hypothalamus.

## Introduction

Heat stress (HS) refers to a series of specific responses to high temperature when the ambient temperature exceeds the upper limit that the animal body can control. In recent years, intensive farming has increased growing density, making birds more susceptible to heat stress as a result of their unique structure (1). The high temperature in summer makes it difficult for the chicken house to cool down, which induces hens suffer from heat stress. Heat stress is mainly manifested as a decrease in animal feed intake, slow body growth, reduced glucose control, lipid metabolism disorders, and decreased immune function (2, 3). Heat stress caused huge economic losses in the poultry industry (4). Hypothalamus is a key brain region that regulates metabolic homeostasis and energy balance, and plays a core role in regulating metabolic functions such as food intake, nutrient metabolism, body water balance, hormone secretion, basic metabolism, body temperature, and circadian rhythm (5). The physiological function of hypothalamus could be damage by heat stress.

In the state of heat stress, the body needs to mobilize the energy reserves of internal organs to fight the high-temperature environment, resulting in destroying metabolic homeostasis (6). Adenosine 5'-monophosphate-activated protein kinase (AMPK) regulates intracellular energy balance and even energy metabolism throughout the body (7). In the hypothalamus, AMPK participates in fatty acid metabolism and regulates food intake and energy metabolism to a certain extent, as well as mediating the effects of peripheral signals (hormones) (8). AMPK activation can regulate appetite promoting and suppressing factors to a certain extent (9). For example, AMPK- aminocyclopropane-1-carboxylate (ACC)- carnitine palmitoyltransferase I (CPT1) pathway can promote fatty acid oxidation and regulate the transcription and expression of appetite promoting peptide agouti-related peptide (AgRP) and neuropeptide y (NPY) (10, 11). However, it remains unclear the correction of heat stress and AMPK signaling pathway and appetite in the hypothalamus.

N-Acetyl-l-cysteine (NAC) was discovered in 1970 and is primarily considered to be an antioxidant. NAC has good antioxidant, immunomodulatory and anti-apoptotic effects in the clinic (12, 13). It has been pointed out that NAC has significant resistance to oxidative stress and inflammatory response caused by heat stress (14). However, it remains unclear whether NAC can effectively improve the energy metabolism disorder or regulate the expression of appetite genes caused by heat stress. Therefore, in this study, we set out to investigated the regulatory mechanism of heat stress on AMPK activity in the hypothalamus of laying hens and the integration of peripheral hormones in energy homeostasis. It also discussed whether N-acetylcysteine (NAC) can effectively relieve heat stress.

## Materials and Methods

### Animals

After 30-day adaptation period, a total of 120 12-weeks-old Hy-Line Brown laying hens were randomly divided into four groups: the control group (Con group), NAC treatment group (Control + NAC group, i.e., CN group), heat-stressed group (HS group) and heat-stress + NAC treatment group (HS + NAC group, i.e., HSN group). The variables were controlled by changing the environmental temperature. The Con group and HS group were given basal diet, CN group and HS + N group were given 1000 mg/ kg NAC. Con and CN groups were fed at a constant temperature (22 ± 1°C), while the room temperature of the HS group and the HS + N group was set to be 36 ± 1°C for 10 h a day (8 am to 6 pm), and 22 ± 1°C during the rest of the time, which means periodic heat stress. Food and water were fed *ad libitum*. The chickens were exposed to 12 h of light per day and the humidity was set to be between 50 and 55%.

### Tissue Sample Collection

On day 7, 14, and 21, hens were prohibited from feeding and drinking for 12 hours before being euthanized. Blood samples were collected and centrifuged at 3,500 rpm for 10 minutes. The extracted serum was stored at −20°C. Hypothalamus tissue was washed with PBS, snap-frozen in liquid nitrogen, and stored at −80°C for subsequent experiments.

### ELISA

Serum triiodothyronine (T3), thyroxine (T4), and corticosterone (CORT) contents were measured using ELISA kit (Beijing Huaying Biotechnology Research Institute, Beijing, China) based on the Quick EIATM ELISA protocol, according to the manufacturer's instructions.

### Real-Time Quantitative PCR

Total RNA was isolated with TransZolUp Reagent (TransGen Biotech, Beijing, China) according to the protocol reported previously (15). In brief, according to the instructions of the kit, cDNA was synthesized using cDNA Synthesis SuperMix Reagent kit (TransGen Biotech, Beijing, China). qRT-PCR was performed using the Light Cycler 96 system according to the protocol. Data were analyzed by the 2^−ΔΔCt^ method. The specific primer sequences were shown in Table 1.

**Table 1:** Gene primer sequences used in quantitative RT-PCR.

**Gene**	**Forward primer (5^**′**^-3^**′**^)**	**Reverse primer (5^**′**^-3^**′**^)**
AgRP	GGAACCGCAGGCATTGTC	GTAGCAGAAGGCGTTGAAGAA
POMC	GTAGCAGAAGGCGTTGAAGAA	CGCTACGGCGGCTTCA
AMPKα1	TCTTGTAGGCGCTTTTGACGAT	ATCTGTCTCGCCCTCATCCT
mTOR	CCACTTCGCTCTTCTTACACCTT	ACTCTGCTAGCAAACGACCC
AKT1	CACAGCAGTTTGGCAAGGTC	CCTTTTGTGGACCCTTCTGC
LKB1	AGAAAATACCGTGGCCTCCA	GCTGACCACCAATGGGACG
ACC	GGCTGGAATGCTGGCGAC	TGGACTGGAAAACGTCTCGG
CPT1	CACAGGTACGCCTTTACCGT	GGAGAACCCAAGTGAAAGTA
		ATGAA
HMGR	TGGAAACGACATAAAGGCAGAA	CTGGGTTTGGTTCTTGTTCA
PPARα	ATTCGGTCTCTGCTTGTTCA	ACGGAGTTCCAATCGC
GPAT	TGTGGAAGGGCTTGTATCGT	TTCCAACACGCGATTTCTGG
GAPDH	TGGCATCCAAGGAGTGAGC	GGGGAGACAGAAGGGAACAG

### Western Blotting

Protein from hypothalamus tissue was extracted using the kit (Wuhan Seville Biotechnology Co., Ltd., Wuhan, Hubei, China), and the protein concentration was measured by the BCA method. The protein samples were separated using SDS-PAGE (10% separation gel and 4% concentration gel) and then transferred to polyvinylidene fluoride (PVDF) membranes (0.45 μm). The membrane was incubated with 5% nonfat milk at room temperature for 2 h before overnight incubation with specific antibodies at 4°C. Then, it was incubated with the corresponding horseradish peroxidase (HRP)-coupled (1:5,000) secondary antibody for 1 h at room temperature. The signal was detected by a chemiluminescence kit (Changjiang Biotechnology Co., Ltd., Nanjing, Jiangsu, China). Finally, digital images were obtained using the Bio-Rad Gel Detection System (Bio-Rad Laboratories, Inc., Hercules, CA, United States), and the results were analyzed using ImageJ (Image processing software).

### Immunofluorescence of AMPK in the Hypothalamus

The dissected hypothalamus was immediately wrapped in silver paper, which was rapidly frozen with liquid nitrogen for 60 s and then embedded with fully frozen brain tissue at optimal cutting temperature (O.C.T.). The frozen sections (8 μm) were placed at room temperature for 5 min, and then fixed with 4% neutral paraformaldehyde. After 10 min, they were sealed with 3% bovine serum and then incubated with primary antibody at 4°C overnight. The cell nuclei marker was DAPI, and the dilution ratio of the AMPK antibody was 1:100.

### Statistical Analysis

Data analysis was conducted using SPSS 17.0 software. GraphPad Prism 7.0 software was used to plot the graphs. Results were presented in the form of mean value ± standard error (SE). ANOVA and t tests were used to make the comparison between groups. *P* > 0.05 indicates that the difference is not statistically significant. *P* < 0.05 indicates that the difference is statistically significant. *P* < 0.01 indicates that the difference is extremely statistically significant.

## Results

### Serum Indexes

**Figure 1:**
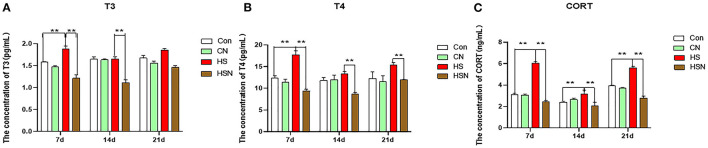
Effects of NAC and heat stress on serum T3, T4 and CORT in layers. **(A)** Serum T3 level. **(B)** Serum T4 level. **(C)** Serum CORT level. *P < 0.01; **P < 0.001.

The influence of NAC on the changes of serum chemical values of hens under heat stress was shown in Figure 1. On day 7 of heat stress, the contents of T3, T4 and CORT in the HS group were significantly upregulated than those in the control group (*P* < 0.01), while, NAC treatment downregulated the contents of T3, T4, and CORT in the HSN group (*P* < 0.01) compared to the HS group. On day 14, the content of CORT in the HS group was still significantly increased (*P* < 0.01). Compared with the HS group, the contents of T3, T4, and CORT in the HSN group were significantly decreased (*P* < 0.01). On day 21, the content of CORT in the HS group were increased than those in the control group (*P* < 0.01). Compared with the HS group, the T4 and CORT of the HSN group were significantly decreased (*P* < 0.01).

### Expressions of Appetite-Related Genes in the Hypothalamus

**Figure 2:**
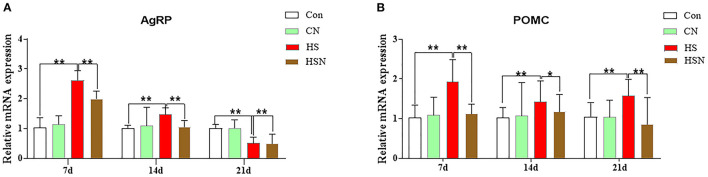
Effects of NAC and heat stress on appetite-related genes expression in the hypothalamus in layers. **(A)** Expression of AgRP mRNA. **(B)** Expression of POMC mRNA. *P < 0.01; **P < 0.001.

Expressions of appetite-related genes in hypothalamus of different groups were shown in Figure 2. On day 7 and 14, compared with the control group, AgRP mRNA expression in the HS group was significantly increased (*P* < 0.01), and decreased in day 21 (*P* < 0.01). Compared with the HS group, the expression of AgRP in the NAC-treated HSN group was significantly decreased (*P* < 0.01 or *P* < 0.05). Compared with the control group, the expression level of proopiomelanocortin (POMC) in the HS group was significantly higher (*P* < 0.01). The POMC expression in the HSN group was significantly lower than that in the HS group (*P* < 0.01 or *P* < 0.05).

### Expressions of AMPK Pathway-Related Genes in the Hypothalamus

**Figure 3:**
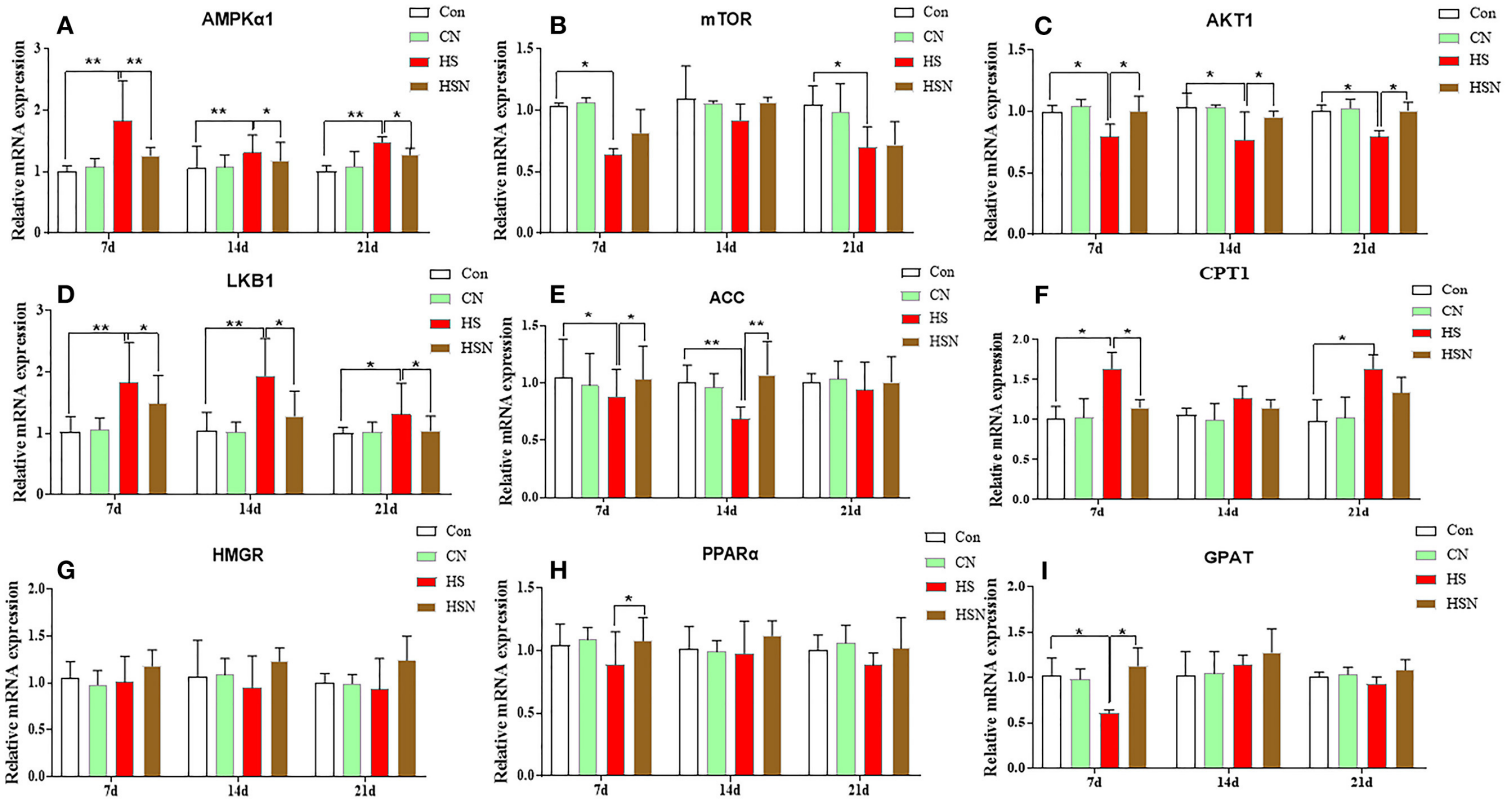
Effects of NAC and heat stress on the mRNA expression levels of AMPK pathway-related genes in the hypothalamus. **(A)** Expression of AMPKα1 mRNA. **(B)** Expression of mTOR mRNA. **(C)** Expression of AKT1 mRNA. **(D)** Expression of LKB1 mRNA. **(E)** Expression of ACC mRNA. **(F)** Expression of CPT1 mRNA. **(G)** Expression of HMGR mRNA. **(H)** Expression of PPARα mRNA. **(I)** Expression of GPAT mRNA. *P < 0.01; **P < 0.001.

Figure 3 showed the effect of NAC on the expression of AMPK pathway-related genes in the hypothalamus under heat stress. On day 7, the mRNA expression of the catalytic alpha subunit of AMPK (AMPKα1), liver kinase b1 (LKB1), and CPT1 in the HS group increased significantly (*P* < 0.05 or *P* < 0.01). The expression of AKT1, ACC, mammalian target of rapamycin (mTOR), and glycerol-3-hosphate acyltransferase enzyme (GPAT) decreased significantly (*P* < 0.05). The expression of 3-hydroxy-3-methyl-glutaryl-coenzyme reductase (HMGR) and peroxisome proliferator-activated receptor α (PPARα) decreased but not significantly (*P* > 0.05). Compared with the HS group, the expression levels of AMPKα1, LKB1, and CPT1 in the HSN group were significantly decreased (*P* < 0.01 or *P* < 0.05). The expression levels of AKT1, PPARα, ACC, and GPAT in the HS group were significantly increased (*P* < 0.05). On day 14, the AMPKα1 and LKB1 expression in HS group increased significantly (*P* < 0.01), and the AKT1 and ACC expression decreased significantly (*P* < 0.01 or *P* < 0.05). After NAC treatment, the AMPKα1 and LKB1 expression in the HSN group decreased significantly (*P* < 0.05), and the AKT1 and ACC expression increased significantly (*P* < 0.01 or *P* < 0.05). On day 21, the AMPKα1, LKB1 and CPT1 expression increased significantly in the HS group (*P* < 0.01 or *P* < 0.05), and the mTOR and AKT1 expression decreased significantly (*P* < 0.05). Compared with the HS group, the expression of AMPKα1 and LKB1 in the NAC-treated HSN group was significantly decreased (*P* < 0.05), and the AKT1 expression was significantly increased (*P* < 0.05).

### AMPK Protein Expression in the Hypothalamus

**Figure 4:**
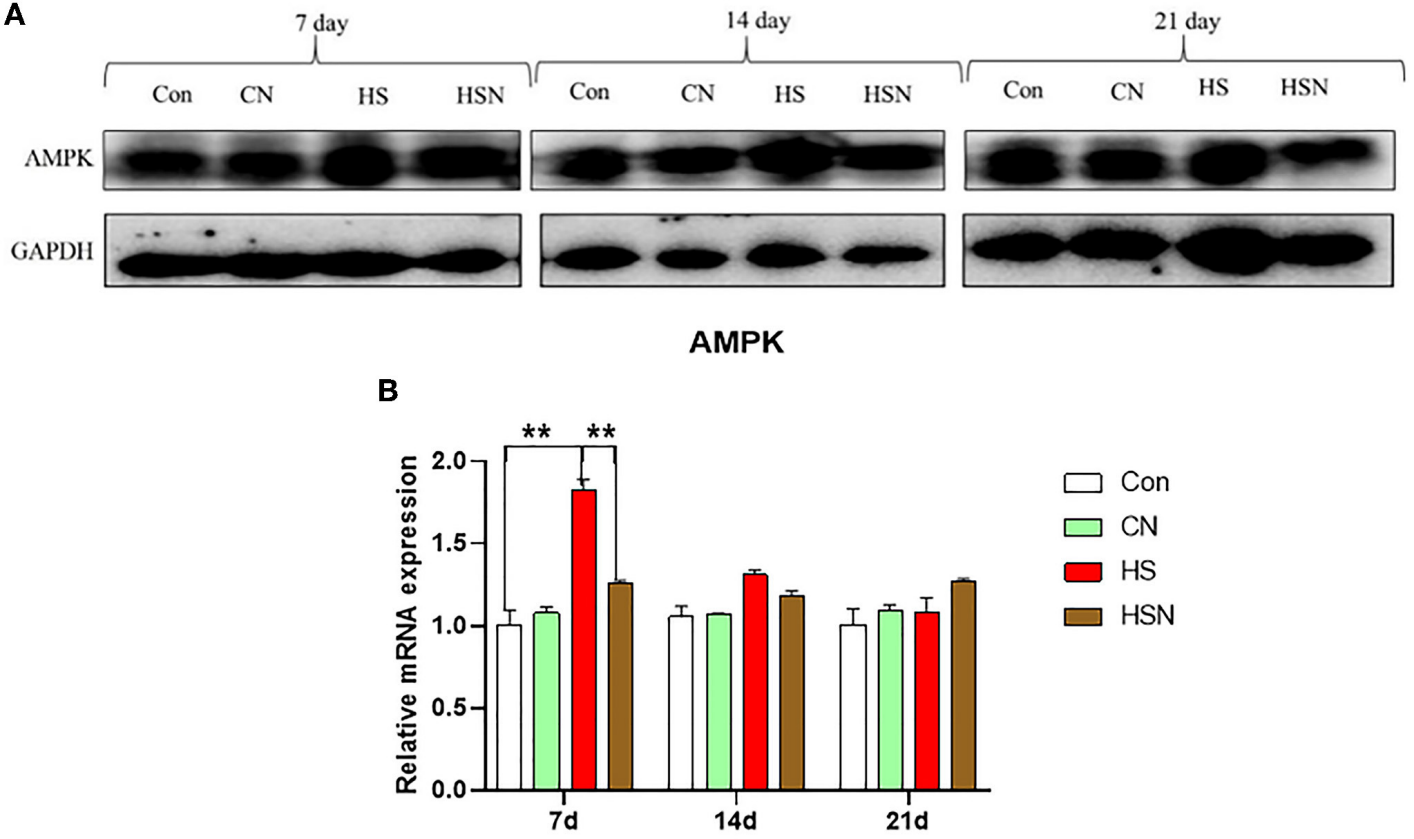
Effects of NAC and heat stress on the expression levels of key proteins in hypothalamus. **(A)** AMPK protein expression levels. **(B)** Relative mRNA expression level of AMPK. *P < 0.01; **P < 0.001.

The grayscale scanning results of AMPK expression level were shown in Figure 4. On day 7, the AMPK protein expression level in control group was significantly lower than that in the HS group (*P* < 0.01), and the AMPK protein expression level in the HSN group was significantly lower than that in the HS group (*P* < 0.01).

### Immunofluorescence Analysis

**Figure 5:**
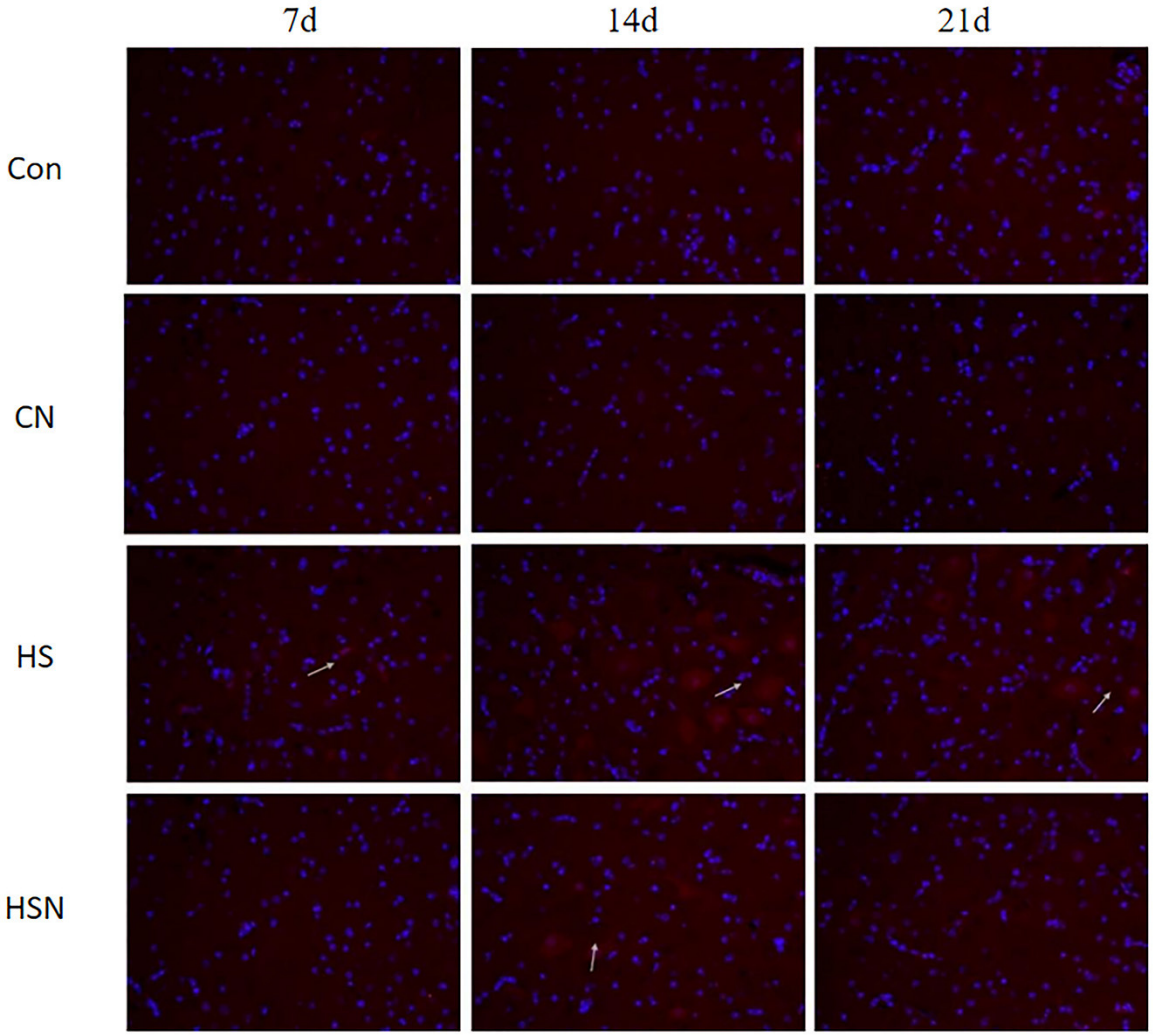
Effect of heat stress and NAC on protein levels of AMPK in the hypothalamus (400 ×) (the red part pointed by the arrow is the AMPK protein).

Figure 5 showed immunofluorescence expression of AMPK in the hypothalamus. On day 14 of heat stress, the AMPK fluorescence intensity was considerably greater in the HS group than in the control group, while NAC dramatically lowered AMPK fluorescence intensity. The white arrow in Figure 5 indicated the positive expression of the AMPK protein.

## Discussion

Heat stress can lead to metabolic disorders, including glucose metabolism disorders, fat metabolism disorders, and hormone metabolism disorders (16–18). Hypothalamus is an important organ with the function of integrating various metabolic regulatory signals. When laying hens suffer heat stress, the hypothalamus can be easily damaged, which changes the metabolism of laying hens and inhibits the performance of laying hens. NAC has been proved to have a certain therapeutic effect on oxidative stress and inflammation induced by heat stress (14). Whether this treatment of injury can effectively regulate the body's metabolic disorders remain unclear. The purpose of this study was to investigate the regulatory role of NAC in energy metabolism disorders induced by heat stress.

Among different metabolic pathways, AMPK has attracted a lot of attention as a measure of systemic energy homeostasis. Activation of AMPK inhibits the anabolic pathway of ATP consumption and induces the catabolic pathway of ATP production (19). These pathways work by regulating gene expression of key metabolic enzymes such as fatty acids, cholesterol, and protein synthesis (20). Studies have shown that the fatty acid biosynthesis pathway includes two key enzymes: ACC and FAS, both of which are expressed in the hypothalamus (21). Malonyl-CoA is not only a mediator of fatty acid biosynthesis but also plays an important role in the control of metabolic conversion between fat production and fatty acid oxidation. AMPK plays an important role in fatty acid biosynthesis. After activation, AMPK phosphorylates and inhibits ACC, prevents malonyl-CoA production, further stimulates CPT1, and ultimately promotes mitochondrial fatty acid oxidation (21). Our results showed that heat stress activated AMPK and inhibited the expression of ACC, which could further stimulate the expression of CPT1. LKB1 is an activation enzyme upstream of AMPK that is responsible for activating energy stress. The LKB1-AMPK signaling pathway promotes fatty acid oxidation by inhibiting glycogen production in response to energy stress. Finally, the results showed that the LKB1 expression in the hypothalamus was significantly increased under heat stress (22). HMGR is an enzyme that affects the differentiation of adipocytes and catalyzes the reduction of 3-hydroxy-3-meglutarate mono-acyl-CoA in cholesterol biosynthesis (23). Activation of AMPK inhibits the expression of HMGR. PPARα is an important gene which is located downstream of AMPK. It is involved in fatty acid metabolism and can maintain cell energy homeostasis (24). The high expression of GPAT can prevent acyl-CoA from entering the mitochondrial matrix, weaken the competition of fatty acid pools, and accelerate the synthesis rate of triglycerides (25). In our results, the changes in the expression levels of these three genes were not significant. In addition, when ATP is depleted, activation of AMPK can lead to inhibition of the mTOR signaling pathway downstream of AMPK, which in turn can inhibit protein synthesis, enabling AMPK to conserve cellular energy even in a low-energy state (26). In addition, mTOR is regulated by AKT, and the activated AKT can directly phosphate mTOR (27). Our results showed that activation of AMPK induced by heat stress could inhibit the expression of mTOR and AKT1. However, after NAC treatment, it could effectively inhibit the abnormal expression of AMPK-related genes induced by heat stress, and make the abnormal expression of genes tend to be normal. This indicated that in our experiment, NAC could effectively inhibit the activation of hypothalamus AMPK caused by heat stress, effectively reduce the decomposition of fatty acids, and maintain the energy metabolism homeostasis of hypothalamus. In addition, it has been found that AMPK acts as a sensor and regulator of energy homeostasis throughout the body, linking the metabolic state with the neuropeptide system and ultimately regulating food intake (28).

The hypothalamus is a key center for regulating appetite and energy balance by regulating the expression of specific neurotransmitters/neuropeptides in response to nutrient/hormone signals, thereby promoting the balance of energy intake and expenditure (29). The center in the hypothalamus that controls food intake consists of two groups of neurons that receive peripheral nutrient signals. On the one hand, they participate in the regulation of feeding through the expression of different appetite peptides, such as AgRP. On the other hand, anti-eating neuropeptides include POMC and so on. As a regulator of energy homeostasis throughout the body, activation of AMPK can stimulate feeding, whereas inhibition of AMPK activity in the hypothalamus can lead to anorexia. Our experimental results showed that the expression of the appetite-promoting AgPR gene was significantly increased under heat stress, while the expression of the appetite-suppressing POMC gene was significantly decreased. The addition of NAC in the diet effectively inhibited the overexpression of AgRP and promoted the expression of POMC. This indicated that NAC effectively inhibited heat stress, mitigated its effects and effectively controlled the expression of appetite genes, and further maintained the normal energy balance of the body.

Heat stress can also stimulate the body's systemic neuroendocrine response to activate the hypothalamic-pituitary-adrenal system and stimulate the secretion of adrenal corticosteroids (30). The secretion rate of adrenocortical hormone is related to basal metabolic rate and heat production (28). The common adrenal corticosteroid is the glucocorticoid CORT. On the one hand, glucocorticoids can directly affect the metabolism of the body. On the other hand, they can directly damage hippocampal neurons through metabolism and other ways (31). Our experimental results showed that heat stress significantly increased the content of CORT in serum, indicating that heat stress activated the hypothalamic-pituitary-adrenal axis and improved the basic metabolism of the body.

## Conclusion

Our results indicated that NAC can effectively ameliorate the pathological changes in hypothalamus caused by heat stress by alleviating the metabolic disorders of T3, T4, and CORT in serum, inhibiting the activation of the hypothalamus AMPK pathway, and regulating the expression of appetite genes.

## Data Availability Statement

The original contributions presented in the study are included in the article/Supplementary Material, further inquiries can be directed to the corresponding authors.

## Ethics Statement

The animal study was reviewed and approved by Animal Health and Utilization Committee of Jiangxi Agricultural University.

## Author Contributions

ZL: data management, research, methodology, and writing—manuscript. YZha: data analysis, investigation, and method. YZhu: visualization. ZX and CW: verification. PL and GH: project management. GL: analysis. WC: software. XGa: correction and editor. XGu: conceptualization, funding access, methods, and supervision. All authors contributed to the article and approved the submitted version.

## Funding

This work was supported by National Natural Science Foundation of China (Nos. 32060760 and 31460679), Natural Science Foundation of Jiangxi Province (No. 2017ACB20012), and the Technology System of Modern Agricultural Poultry Industry of Jiangxi Province (JXARS).

## Conflict of Interest

The authors declare that the research was conducted in the absence of any commercial or financial relationships that could be construed as a potential conflict of interest.

## Publisher's Note

All claims expressed in this article are solely those of the authors and do not necessarily represent those of their affiliated organizations, or those of the publisher, the editors and the reviewers. Any product that may be evaluated in this article, or claim that may be made by its manufacturer, is not guaranteed or endorsed by the publisher.
