# Supplementary material for: Effects of N-Acetyl-L-Cysteine on Serum Indices and Hypothalamic AMPK-Related Gene Expression Under Chronic Heat Stress
Source: Front Vet Sci. 2022 Jun 15;9:936250. doi: 10.3389/fvets.2022.936250 (PMC9242840; doi:10.3389/fvets.2022.936250)
Supplement: Supplementary file 3 [file Data_Sheet_3.docx]

**Supplementary Material Captions**

Sheet 1: The original data of T3,T3 and CORT. R represents HS group, RN represents HSN group, Z represents Con group and ZN represents CN group. 1- represents 7 days, 2- represents 14 days, and 3- represents 21 days.

Sheet 2: Table 1 shows the results of QPCR, with 4 samples for each gene and 3 repeat holes for each sample. R represents HS group, RN represents HSN group, Z represents Con group and ZN represents CN group. 1- represents 7 days, 2- represents 14 days, and 3- represents 21 days.

Image 1 shows the bands of AMPK protein and GAPDH.

Image 2 shows immunofluorescence of AMPK in different groups at different periods.
