# Supplementary figures and images for: Effects of N-Acetyl-L-Cysteine on Serum Indices and Hypothalamic AMPK-Related Gene Expression Under Chronic Heat Stress
Source: Front Vet Sci. 2022 Jun 15;9:936250. doi: 10.3389/fvets.2022.936250 (PMC9242840; doi:10.3389/fvets.2022.936250)

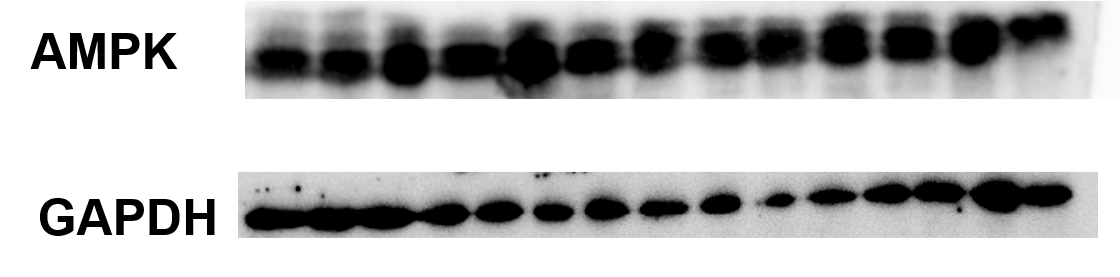

Supplement: Supplementary file 4 [file Image_1.TIF]

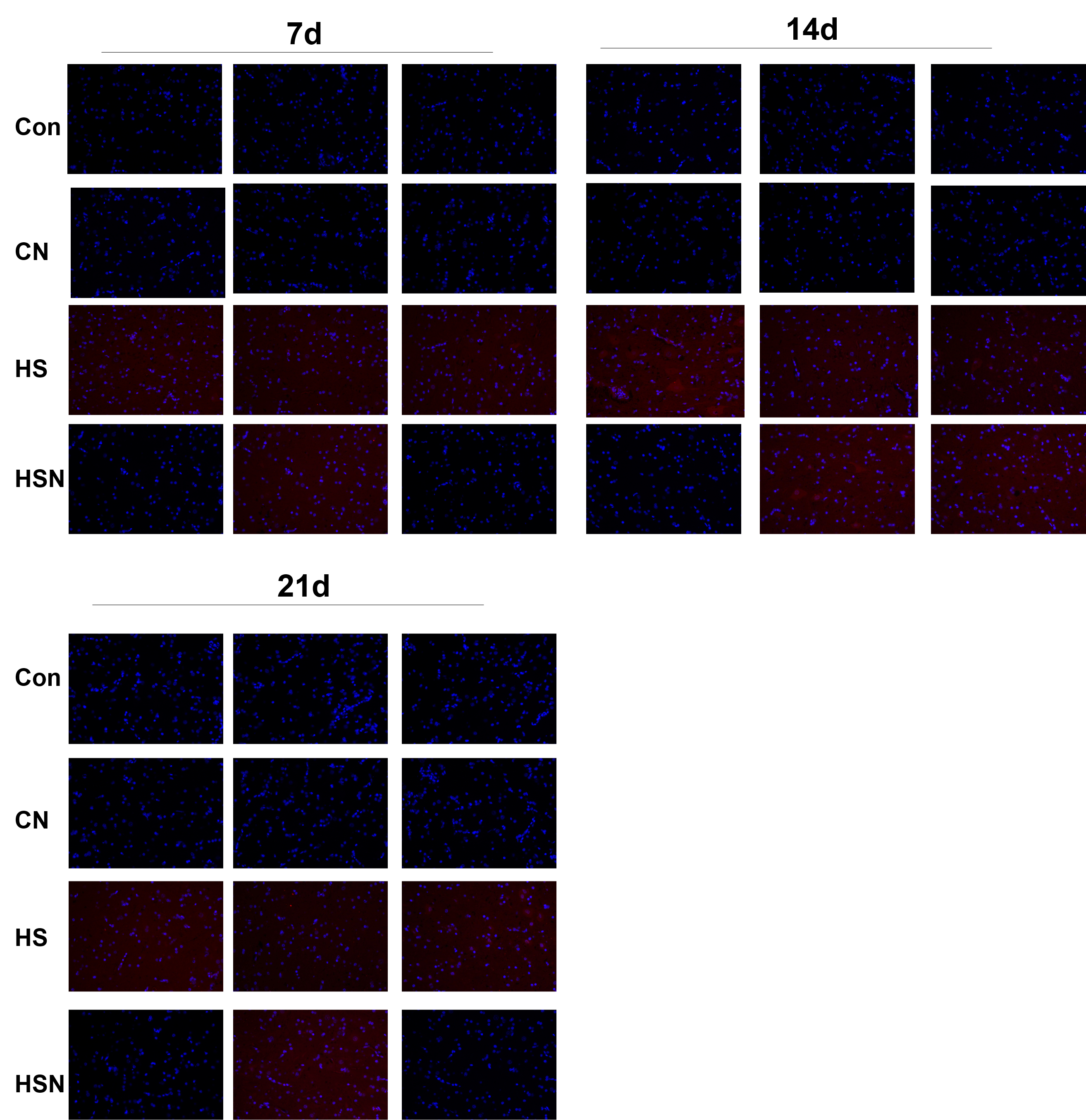

Supplement: Supplementary file 5 [file Image_2.TIF]
